# Detection of bacterial and protozoan pathogens in individual bats and their ectoparasites using high-throughput microfluidic real-time PCR

**DOI:** 10.1128/spectrum.01531-23

**Published:** 2023-08-22

**Authors:** Alexandra Corduneanu, Zbigniew Zając, Joanna Kulisz, Aneta Wozniak, Angélique Foucault-Simonin, Sara Moutailler, Alejandra Wu-Chuang, Áron Peter, Attila D. Sándor, Alejandro Cabezas-Cruz

**Affiliations:** 1 Department of Animal Breeding and Animal Production, University of Agricultural Sciences and Veterinary Medicine, Cluj-Napoca, Romania; 2 Department of Parasitology and Parasitic Diseases, University of Agricultural Sciences and Veterinary Medicine, Cluj-Napoca, Romania; 3 Department of Biology and Parasitology, Medical University of Lublin, Lublin, Poland; 4 ANSES, INRAE, Ecole Nationale Vétérinaire d’Alfort, UMR BIPAR, Laboratoire de Santé Animale, Maisons-Alfort, France; 5 Department of Parasitology and Zoology, University of Veterinary Medicine, Budapest, Hungary; 6 ELKH-ÁTE Climate Change: New Blood-sucking Parasites and Vector-borne Pathogens Research Group, Budapest, Hungary; University of São Paulo, São Paulo, Brazil

**Keywords:** Bats, piroplasms, bacterial pathogens, high-throughput screening

## Abstract

Among the most studied mammals in terms of their role in the spread of various pathogens with possible zoonotic effects are bats. These are animals with a very complex lifestyle, diet, and behavior. They are able to fly long distances, thus maintaining and spreading the pathogens they may be carrying. These pathogens also include vector-borne parasites and bacteria that can be spread by ectoparasites such as ticks and bat flies. In the present study, high-throughput screening was performed and we detected three bacterial pathogens: *Bartonella* spp., *Neoehrlichia mikurensis* and *Mycoplasma* spp., and a protozoan parasite: *Theileria* spp. in paired samples from bats (blood and ectoparasites). In the samples from the bat-arthropod pairs, we were able to detect *Bartonella* spp. and *Mycoplasma* spp. which also showed a high phylogenetic diversity, demonstrating the importance of these mammals and the arthropods associated with them in maintaining the spread of pathogens. Previous studies have also reported the presence of these pathogens, with one exception, *Neoehrlichia mikurensis*, for which phylogenetic analysis revealed less genetic divergence. High-throughput screening can detect more bacteria and parasites at once, reduce screening costs, and improve knowledge of bats as reservoirs of vector-borne pathogens.

**IMPORTANCE:**

The increasing number of zoonotic pathogens is evident through extensive studies and expanded animal research. Bats, known for their role as reservoirs for various viruses, continue to be significant. However, new findings highlight the emergence of *Bartonella* spp., such as the human-infecting *B. mayotimonensis* from bats. Other pathogens like *N. mikurensis*, *Mycoplasma* spp., and *Theileria* spp. found in bat blood and ectoparasites raise concerns, as their impact remains uncertain. These discoveries underscore the urgency for heightened vigilance and proactive measures to understand and monitor zoonotic pathogens. By deepening our knowledge and collaboration, we can mitigate these risks, safeguarding human and animal well-being.

## INTRODUCTION

Bats are the only mammals that can actively fly. There is a large number of species distributed worldwide and divided into two suborders: Yinpterochiroptera and Yangochiroptera (1). The bat species found in Europe and especially in Romania belong to both suborders (2, 3). Due to their life history, behavior, foraging, and ability to migrate over long distances, they can be the “perfect” reservoirs for various pathogens (4). Several viral diseases that affect humans and livestock have been linked to bats as reservoirs (5 – 7). Bacterial and parasitic diseases are also known to be transmitted from these mammals to humans or domestic animal populations, with possible effects on their health (8 – 12). Among these, the genus *Bartonella* spp. is a Gram-negative facultative intracellular α-proteobacterium found in mammalian erythrocytes and endothelial cells (13). While some species are known to be pathogenic to humans (e.g., *Bartonella henselae*, *B. quintana*, *B. grahamii*, and *B. washoensis*) (14 – 19), others are newly described and their pathogenicity is still unclear (e.g., *B. mayotimonensis*) (20). The exact role of bats as carriers or reservoirs of different species of *Bartonella* is still unclear and undetermined, but this role is being studied very intensively. This bacterial pathogen is the most studied in various samples collected from bats: tissues (21, 22), ticks (23, 24), bat flies (25 – 28), and bat bugs (29). A newly described species of *Bartonella—*“*Candidatus* B. mayotimonensis,” which has been associated with cases of endocarditis in human patients from the USA (30) was also isolated from bat samples collected in Europe (20, 22, 31).

Due to the growing number of people in the world and their close contact with animals, more and more pathogens with a possible zoonotic potential are being described. This is also the case with *Neoehrlichia mikurensis*, a pathogen that is transmitted by ticks of the genus *Ixodes*, especially *I. ricinus* (32, 33). This bacterial pathogen was first found in the spleen of rats from Japan and in *I. ovatus* ticks (32). Later, the first evidence was documented in a human patient in Sweden, followed by other European countries (33, 34). Studies on the epidemiology and detection of this bacterium reported its presence in various species of hard ticks, like *Ixodes* spp. (35 – 39), in *Dermacentor reticulatus* (38), in wildlife (40 – 43), and in domestic animals (38, 39, 44, 45), and also in humans (46 – 48). The main reservoirs for *N. mikurensis* are probably various rodent species (49). There are no studies reporting the presence of this bacterial pathogen in bat species.

Hemotropic mycoplasmas, also called hemoplasmas, are Gram-negative bacteria of the genus *Mycoplasma*, found on the surface of mammalian erythrocytes and are transmitted by contact with droplets of nasal and oral secretions (50). There are studies of the occurrence of different *Mycoplasma* species in wild animals (51, 52), domestic animals (53), and humans with varying degrees of pathogenicity (54). The first report of *Mycoplasma* in bats comes from the little brown bat (*Myotis lucifugus*) from the USA (55). Later, various studies reported the presence of mycoplasmas in bats from Africa (56), Asia (57), Central and South America (58 – 61), and Europe (62, 63). Sequences of mycoplasmas isolated from bats in Spain showed that they are closely related to the species “*Candidatus* Mycoplasma hemohominis” (64), which has also been isolated from humans in different parts of the world (65 – 68).

Protozoan parasites (e.g., *Babesia* spp. and *Theileria* spp.) are emerging tick-borne zoonotic pathogens (69, 70). They are one of the most widespread blood parasites in the world after trypanosomes and malaria and have significant economic, medical, and veterinary implications (71 – 75). In bats, the first report of protozoan parasites (e.g., *Babesia vesperuginis*) was published in Italy in 1898, followed by several studies describing a similar parasite in various bat species (76 – 80). Different species of *Babesia* and *Theileria* were already detected in bat-specific ticks (e.g., *I. ariadnae*, *I. vespertilionis,* and *I. simplex*) collected in Hungary and Romania (81).

Vector-borne bacterial and parasitic pathogens are usually detected using molecular biology [conventional PCR (cPCR), nested PCR (nPCR), or reverse transcription PCR (RT-PCR)], in which one or more genes are targeted to assess the presence or absence of that specific pathogen. Although these methods are most commonly used, they are time-consuming and resource-intensive. A new approach is microfluidic analysis, in which multiple pathogens targeting one or more genes can be determined at once (82 – 85). Considering the advantages of this analysis, the objectives of the present study were the following: (i) high-throughput screening of pathogens, (ii) pairing bat-arthropod samples, and (iii) phylogenetic and haplotype divergence analysis of commonly occurring pathogens in bats and their parasites.

## RESULTS

### Detection of pathogens in blood collected from insectivorous bats

A total of 48 blood samples from two different bat species (*Miniopterus schreibersii*: *n* = 39 and *Myotis capaccinii*: *n* = 9) were analyzed by high-throughput microfluidic real-time PCR. For the presence of four pathogens, the positive samples for RT-PCR were validated by cPCR or nPCR (Table S1). All pathogen groups tested were detected, as follows: Apicomplexa (2.08% of the total number of blood sample tested), *Bartonella* spp. (16.66%), *Neoerlichia mikurensis* (10.41%), and *Mycoplasma* spp. (12.5%) (Table 2). Samples from the bat species *M. schreibersii* were positive for all four pathogens, while samples from *Myotis capaccinii* were positive only for *Bartonella* spp. (Table 1).

**TABLE 1:** Detection of pathogens in bat samples

Type of sample	Pathogen detected	Bat species	Ectoparasite/development stage	Location	Accession numbers
Host tissue	Apicomplexa	*Miniopterus schreibersii*	-	Galeria din Pădure-Canaraua Fetii	OQ255847
	*Neoerlichia mikurensis*	*Miniopterus schreibersii*	-	Baziaş; Peştera Liliecilor; Gura Dobrogei; Galeria din Pădure- Canaraua Fetii	OP999371– OP999375
	*Bartonella* spp.	*Miniopterus schreibersii*	-	Baziaş; Peştera Liliecilor; Gura Dobrogei; Ineu	OQ054993– Q054994 OQ055003– Q055004 OQ055009 OQ055014
		*Myotis capaccinii*	-	Baziaş	OQ054995– OQ054996
	*Mycoplasma* spp.	*Miniopterus schreibersii*	-	Baziaş; Galeria din Pădure-Canaraua Fetii	OQ274904 - OQ274909
Bat flies	*Bartonella* spp.	*Miniopterus schreibersii*	*Nycteria schmidlii*/ adult	Baziaş; Peştera Liliecilor; Gura Dobrogei; Galeria din Pădure-Canaraua Fetii	OQ054997– OQ055002 OQ055005– OQ055008 OQ055010– OQ055013
		*Myotis capaccinii*	*Nycteria pedicularia*/ adult	Baziaş	OQ054997 OQ055006 OQ055007
Ticks	*Mycoplasma* spp.	*Miniopterus schreibersii*	*Ixodes simplex*/larva	Galeria din Pădure- Canaraua Fetii	OQ274910

**TABLE 2:** Number of positive samples for four pathogens

Pathogens detected	Type of sample	No. of positive samples by high-throughput microfluidic PCR	No. of positive samples by confirmatory PCR/total no. of sample	Prevalence for each pathogen following confirmatory PCRs	No. of positive paired-blood ectoparasites’ (bat flies, ticks) samples
*Bartonella* spp.	Blood	18	8/48	16.66%	3
	Bat flies	36	12/80	15%	
*Anaplasma* spp. (*N. mikurensis*)	Blood	7	5/48	10.41%	-
*Mycoplasma* spp.	Blood	23	6/48	12.5%	1
	Tick	5	1/80	2.08%	
Apicomplexa	Blood	31	1/48	2.08%	-

### Detection of pathogens in ectoparasites collected from bats (ticks and bat flies)

By high-throughput microfluidic real-time PCR, 80 ectoparasites were analyzed (ticks: *n* = 20 and bat flies: *n* = 60) (Table S1). All ticks belonged to a single species, *Ixodes simplex*, the host-specific parasite of *M. schreibersii* (86), while nycteribiids belonged to three species, *Nycteribia pedicularia* (specific parasite of *My. capaccinii*)*, Nycteribia schmidlii,* and *Penicidillia conspicua* [both species-specific parasites of *M. schreibersii* (87)]. The positive samples were paired with those from the blood that were positive and analyzed further. From the same four pathogens tested, only two were detected in the respective host-and-ectoparasite pairs: *Bartonella* spp. was present in two bat fly species (*Nycteribia pedicularia* and *Nycteribia schmidlii*) and their respective host, while *Mycoplasma* spp. was detected in a single tick larva (*I. simplex*) and its hosts (an *M. schreibersii* individual from Canaraua Fetii; Table 2).

### Phylogenetic analysis

Blasting of obtained sequences of 16S rRNA of Anaplasmataceae against National Center for Biotechnology Information GenBank database showed their similarity to *N. mikurensis* and were clustered together with sequences reported from European counties, for example, Poland (KJ123754), Slovakia (KJ649323), Slovenia (KJ408793) and also from the Far East, South Korea (MF351962) (Fig. S1). Sequenced sample of Apicomplexa obtained in this study should be recognized as *Theileria orientalis* and showed high similarity of nucleotide position to other *T. orientalis* sequences reported from China (MH208641), Austria (AB520955), and Turkey (OM066208) (Fig. S2). In the case of *Mycoplasma* 16S rRNA gene, sequenced samples clustered together with *Mycoplasma* spp. sequences reported from Hungary (MH383150 and MH38315) and Spain (KM538698 and KM538692) (Fig. S3). *Bartonella* sequences obtained in the current study were clustered together with sequences of *Bartonella* spp. (e.g., MF288131 and KY232247), *B. washoensis* (e.g., AB674225 and MH547360), and *B. henselae* (e.g., MT095055 and KC422265) (Fig. S4).

**Fig 1:**
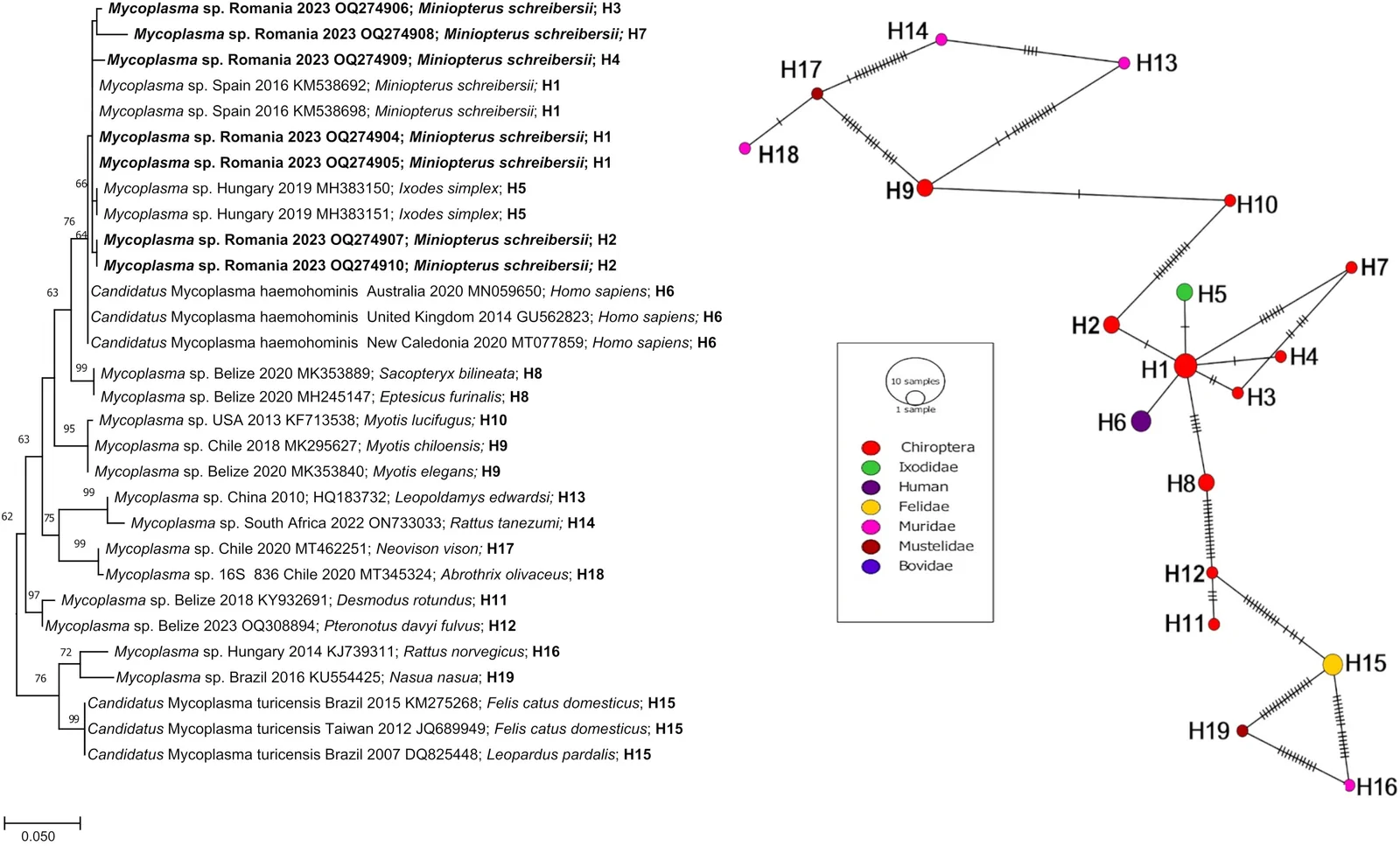
Phylogeny and genotype analysis of Anaplasmataceae inferred from 16S rRNA depending on host reservoir species. The evolutionary history was inferred by using the maximum likelihood method and the Tamura Nei-parameter model. The analysis contains sequences identified in the current study (bold) and GenBank sequences. Accession numbers of sequences are given. Bootstrap values are represented as percentage of internal branches (500 replicates), and values lower than 60 are hidden. The tree is drawn to scale, with branch lengths measured in the number of substitutions per site. This analysis involved 33 nucleotide sequences. There were 406 positions in the final data set. H, haplotype. The diagonal lines indicate the number of mutations between the haplotypes.

**Fig 2:**
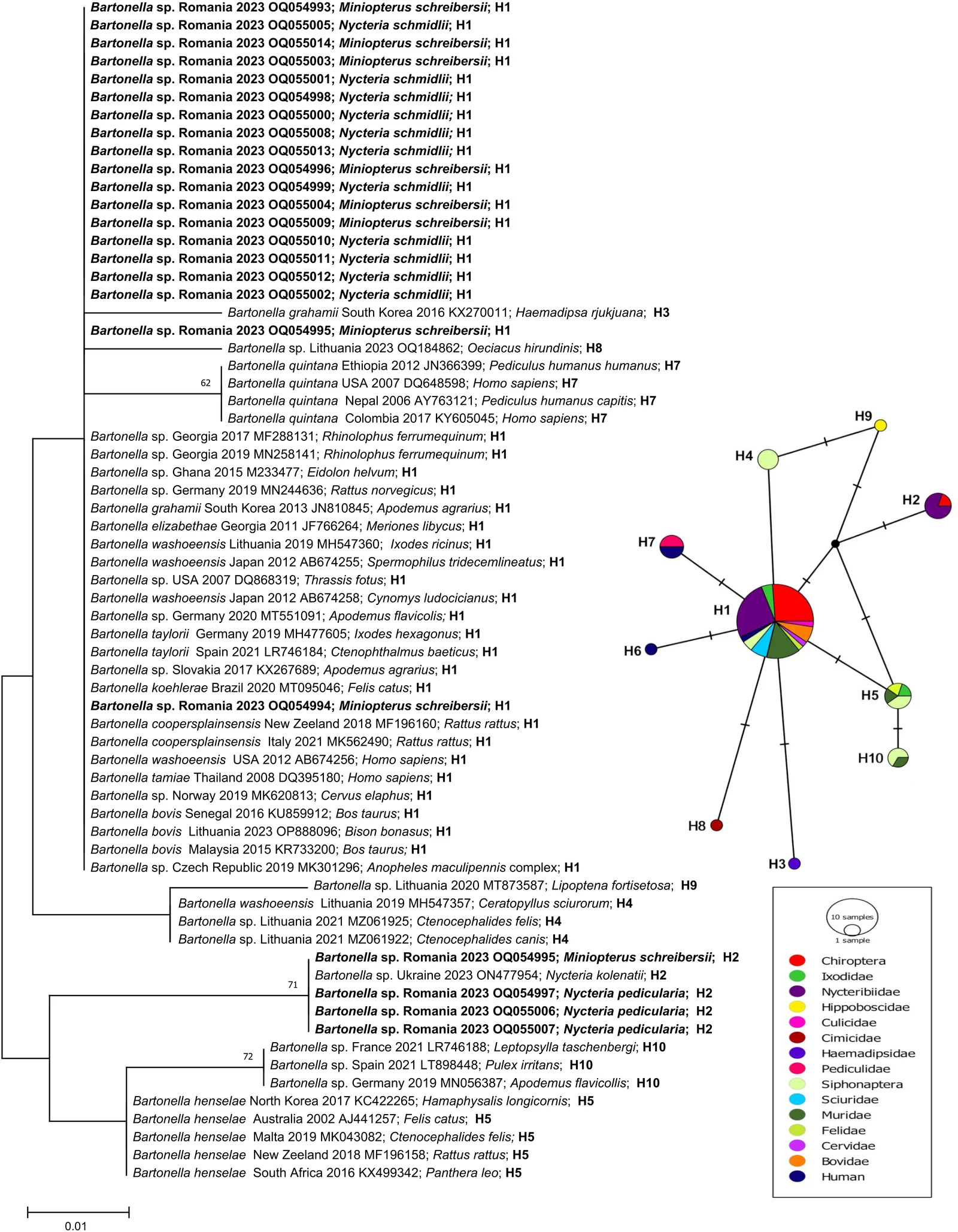
Phylogeny and genotype analysis of *Bartonella* spp. inferred from 16S rRNA depending on host reservoir species. The evolutionary history was inferred by using the maximum likelihood method and the Kimura 2-parameter model. The analysis contains sequences identified in the current study (bold) and GenBank sequences. Accession numbers of sequences are given. Bootstrap values are represented as percentage of internal branches (500 replicates), and values lower than 60 are hidden. The tree is drawn to scale, with branch lengths measured in the number of substitutions per site. This analysis involved 66 nucleotide sequences. There were 74 positions in the final data set. H, haplotype. The diagonal lines indicate the number of mutations between the haplotypes.

Of all the bacteria/piroplasms sequenced in the current study, *N. mikurensis* had the lowest genetic divergence. All sequenced *N. mikurensis* samples belong to the same haplotype (H1). Moreover, the same haplotype was found in other sequences downloaded from GenBank derived from different groups of organisms like mammals (Chiroptera and Rodentia) or arthropods (different Ixodidae species) (Fig. S5). Phylogenetic and genotype analyses of other sequenced samples confirmed that they may circulate between several reservoir and vector organisms. *Theileria orientalis* is most often found in Chiroptera, Bovidae, and Ixodidae (Fig. S6). *Mycoplasma* spp. (Fig. 1) and *Bartonella* spp. (Fig. 2) are characterized by high diversity inside respective reservoir organism groups; however, most often circulate between Chiroptera, Ixodidea, Felidae, and Nycteribiidae (Fig. 1 and 2).

## DISCUSSION

Our aim was to pair positive host-derived blood samples with positive samples of ectoparasites (ticks and bat flies) to determine the reservoir/vectorial role and maintenance routes of pathogens between these two. In the present study, high-throughput real-time microfluidic PCRs were used to detect various bacterial and protozoan pathogens in the blood and ectoparasites of bats from Romania. Phylogenetic analysis was also performed to assess the phylogenetic and haplotype divergence analysis of the pathogens detected. By confirmatory PCRs, we detected the presence of three bacterial pathogens and one protozoan parasite. While *Bartonella* (88), *Mycoplasma* (64), and *Theileria* (81) have already been detected in bat samples, this is, as far as we know, the first report of *N. mikurensis* in the blood of insectivorous bats in Europe.

Due to the great diversity of bat species, the different roosts, and the types of diet, several studies aimed to detect various pathogens, especially *Bartonella*. Various species and strains of *Bartonella* spp. have been detected from the blood (88, 89), tissue (21, 90), and ectoparasites (91, 92) of bats around the world. The molecular methods used to detect these bacteria are generally cPCR and quantitative PCR, targeting especially the *gltA* gene (better detection of genotype diversity and the number of sequences available in GenBank is higher) (78, 93, 94), the *rpoB*, *ftZ,* and *nuoG* genes, alone or in the combination of several genes (95, 96). By confirmatory PCRs, we targeted the ITS gene, which also was used in bat samples (97 – 99). As Stuckey et al. (22) have shown, the prevalence of *Bartonella* infection in bat samples (blood or tissue) can vary between different bat families, with the highest prevalence for insectivorous bats found in the family Miniopteridae (up to 54%). In our study, of the eight positive samples by confirmatory PCRs, six belonged to the bat species *M. schreibersii* and two to the bat species *My. capaccinii*. This confirms a higher prevalence for the family Miniopteridae, which was also observed in a study from Japan, South Africa, and Swaziland (100). Regarding the type of diet, hematophagous or carnivorous bats harbor more *Bartonella* strains and species than bats with other diets (101 – 104) and may maintain infection or transmit different *Bartonella* strains between different vertebrate hosts (102). Since we only have insectivorous bats in Europe, the probability of becoming infected with *Bartonella* is higher than in Africa or America, as it was suggested previously (105). All known *Bartonella* species are transmitted between hosts by different arthropods (15, 106, 107). This bacterium was initially considered an endosymbiont of arthropods and is thought to maintain a commensal relationship with its arthropod host. Bats have been shown to be an important reservoir for *Bartonella* and they probably have evolved together (108). This is also supported by the phylogenetic analysis of the current study (Fig. 2). Regarding the occurrence of this bacterial pathogen in bat ectoparasites (bat flies, fleas, mites, ticks) (105, 107), studies have determined the prevalence and phylogenetic relationships between them and bats. As for bat flies collected and tested especially in Europe, a high prevalence of *Bartonella* strains was found mainly in the *N. schmidlii*, *N. kolenatii*, *N. pedicularia,* and *P. conspicua* (28, 109, 110). This is also confirmed by phylogenetic analyses (Fig. 2). We also identified different strains of *Bartonella* in two bat fly species (*N. schmidlii* and *N. pedicularia*) collected from two insectivorous bat species (*M. schreibersii* and *My. capaccinii*) at three sites in Romania. Both Nycteribia species are highly specialized and host-specific, thus they likely will transmit individual *Bartonella* spp. strains, too. Our results confirmed this hypothesis, as we found a single, but ectoparasite/host-specific strain both in the samples of the two different flies, as well as in the blood samples of their respective hosts (see Fig. S4). The diversity and similarity of *Bartonella* strains have already been demonstrated in bats and their bat flies (92, 98), with stains that can be very specific to bats or their respective bat flies only (21). In the present study, paired bat-arthropod-positive samples were found in three cases: two in *My. capaccinii* and its fly *N. pedicularia*, collected in Baziaş and one in *M. schreibersii* and the bat fly *N. schmidlii*, collected in Gura Dobrogei. Various strains isolated from bats were closely related to strains that can infect humans or rodents, as follows: *B. mayotimonensis* (20, 22, 31, 91), *B. tamiae*, *B. baciliformis* (97, 111, 112), *B. rousetti* (98), *B. washoensis* (113), or *B. schoenbuchensis* (102). The transmission routes of *Bartonella* strains between bats, their ectoparasites, and other hosts are still unknown. Bat flies, which are thought to be the main vector, do not parasitize on humans or other vertebrates, but it has been suggested that direct contact with bats or with their excretions (urine, saliva, feces) may be a possible route of *Bartonella* transmission (31, 114, 115). Furthermore, in countries where bats are consumed, or where contact is very close and physical interspecific interactions (bites or scratches) may occur, host-switches can happen (98).

Vector-borne diseases are constant and emerging problems in human and livestock health, recognized under the “One Health” paradigm (116, 117). In recent years, a number of new, different pathogens have been described in both animals and humans (118). This is also the case with the bacteria *N. mikurensis*, a new candidate from the family Anaplasmataceae, which was first described from rodents (*Rattus norvegicus*) and ticks (*Ixodes ovatus*) and assessed with unknown pathogenicity (32). Later, in 2010, there were case reports of the presence of *N. mikurensis* in human patients from Sweden, Germany, Switzerland, and China (19, 33, 48, 119). In Europe, this pathogen is transmitted mainly by the ubiquitous tick, *Ixodes ricinus* (120), the main vector for numerous vector-borne diseases (121), while in Russia it has been detected in *I. persulcatus* and a rodent host, *Apodemus peninsulae* (122, 123). The prevalence of infection with *N. mikurensis* in Europe varies from 0.08% (*Dermacentor reticulatus*) (124) to 100% in *Ixodes* spp. (likely *I. ricinus* larvae) (49). Various species of rodents are considered to be the main reservoir for this pathogen (37), but the natural cycle, geographical distribution, host reservoirs, or pathogenicity are still unclear and undetermined. In our study, we have detected the presence of a bacterium from the family Anaplasmataceae in the blood of the bat species *M. schreibersii*, but the route of infection is unknown. The most likely way is transmission by *I. ricinus*, which is a generalist tick and common parasite of many rodent species, but also bats. While it is rare on bats, it was already recorded as bat-parasites in several instances (86). Our samples showed a 99% identity with sequences isolated from different rodent species, ticks, and humans. From a phylogenetic point of view, *N. mikurensis* forms its own cluster within the family Anaplasmataceae, which also includes *Ca* Neoehrlichia lotoris (125). Our analysis shows a possible reservoir role of bats in maintaining *N. mikurensis* in the environment (Fig. S5).

One of the smallest organisms discovered in bats and their ectoparasites is *Mycoplasma* spp., of which several species have been described in mammals, humans, reptiles, fish, arthropods, and even plants (126). Because bats can harbor a variety of microorganisms that have little or no effect on their health, studies have focused on the detection of those organisms which may have a zoonotic importance, including the detection of these mollicutes in different types of samples collected from them. High prevalence of hemoplasmas have been found in the blood or tissue of different bat species, and the prevalence was ranging from 3.2% (62) to 97% (64). In Europe, there are two studies reporting the presence of *Mycoplasma* spp. in bats, particularly in the species *M. schreibersii*: the present study, which found a prevalence of 12.5%, and another from Spain (64), showing a much higher (97%) prevalence. The differences in the prevalence of infection between these studies may be due to different bat species, different habitats, different diets, and the genetic diversity of hemoplasmas present. While a higher prevalence of *Mycoplasma* spp. has been observed in bats in general, a lower prevalence has been reported in bat-associated ectoparasites (e.g., bat flies, ticks, and mites) (57, 63). These bacteria were previously detected in *I. simplex* collected from bats in China (57) (prevalence of 3.37%) but also in Hungary [prevalence of 0.7% (63)]. From the same bat tick (*I. simplex*), we had a single positive larva collected from a positive bat for *Mycoplasma* spp., and these results extend the knowledge of the geographical range of this pathogen in Europe. Phylogenetic analysis of the hemoplasma sequences revealed that those isolated from bats in Africa and Central and South America are similar among each other, while several different genotypes are circulating in these regions (56, 59, 61, 62). The genotypes isolated from bats and their ectoparasites from USA, Spain, and Hungary cluster together with *Ca*. M. hemohominis and *Ca*. M. hemomuris (55, 63, 64).

Most studies of intracellular parasites of bats focus on protozoan parasites, with an emphasis on piroplasms and hemosporidians (127, 128), especially in insectivorous bats (12, 79). While *Babesia* spp. has been described in bats in 1898 and has been identified as *Babesia vesperuginis* in several studies (12, 77, 78), *Theileria* spp. has only been detected in a few bat-derived samples. The first evidence of piroplasms in frugivorous bats was recorded in *Pteropus rufus* from Madagascar, where a prevalence of 4.43% was recorded (129). Later, another study showed a prevalence of 12.6% in frugivorous and omnivorous bats from Brazil, with the detected pathogens characterized as “Piroplasmid n. sp.” (from *Phyllostomus discolor*) and “Piroplasms sp.” (recorded from *Artibeus* spp. bats) (80). The only study reporting the presence of *Theileria* spp. (e.g., *T. capreoli*, *T. orientalis,* and *Theileria* sp. OT3) in ticks (larvae and females of *Ixodes simplex*) collected from insectivorous bats from Europe was conducted by Hornok et al. (81). The route of transmission and presence of these *Theileria* species is thought to be due to the tick *Haemaphysalis* spp. which happened to infest bats, too (81). In the present study, using confirmatory PCRs we detected only one positive sample for Apicomplexa targeting the 18S rRNA gene, which after sequencing proved to be *Theileria* spp. Our sequences clustered together with nine sequences of the 18S rRNA gene from Madagascar showed high similarity with other sequences from rodents, primates, and canid babesia, all belonging to the “*microti* group” (129), while the eight amplicon sequences analyzed from Brazil showed high identity with *Theileria bicornis* and three showed high identity with *Babesia vogeli* (80).

Due to the great diversity of bat species and their ectoparasites (ticks and bat flies), numerous pathogens (bacteria and parasites) can be spread among them and also among other animals and humans. Overall, in our study four pairing bat-arthropod samples were detected to be positive for two different bacteria: *Bartonella* spp. and *Mycoplasma* spp., which were recorded in high prevalences both in bats and their respective ectoparasites. In addition, here we present the first records of *N. mikurensis* and an undescribed *Theileria* spp. in the bat blood, thus providing further evidence in support of the role bats and their ectoparasites play in their respective epidemiology. In this ground-breaking study, we have advanced pathogen screening by employing high-throughput microfluidic real-time PCR for the first time on bat samples and paired bat-arthropod samples. This technique allows comprehensive screening of diverse pathogens, significantly reducing analysis time and advancing our understanding of vector-borne diseases in bats.

## MATERIALS AND METHODS

### Sample collection

Bat samples [blood (*n* = 48) and ectoparasites (*n* = 80)] were collected from four different locations in Romania: Baziaş, Canaraua Fetii, Ineu, and Gura Dobrogei, in autumn 2021 and spring 2022. Mist nets and harp traps were used to capture the bats, and these were placed close to the entrance to their roosts. The bats were identified to species level using the available keys (3), and age, sex, forearm length, and body weight were recorded for each individual. In the present study, samples were collected from two insectivorous bat species: *M. schreibersii* [blood (*n* = 39), ticks (*n* = 19), and bat flies (*n* = 50)] and *My. capaccinii* [blood (*n* = 9), ticks (*n* = 1), and bat flies (*n* = 10)]. The difference between the numbers of samples collected from the two species resulted from the abundance of the bat species *M. schreibersii* as well as the presence of several ectoparasites and a high number in this particular species.

A drop of blood was taken from each bat by using venipuncture applied to the right saphenous (interfemoral) vein. Each sampled bat was physically immobilized, the uropatagium was disinfected with alcohol, and blood was taken from the uropatagial vein with 28-gauge needle. The blood was transferred onto the filter paper and then stored individually in a sterile tube. Each tube was individually labeled and stored at 4°C until DNA extraction. Bat ectoparasites (ticks—Ixodoidea: Ixodidae and bat flies—Diptera: Nycteribiidae) were also collected from the same individuals and stored in sterile tubes (type of ectoparasite/each bat) with 70% ethanol until further identification.

#### Ectoparasite identification

Identification of each ectoparasite was based on morphological keys (130, 131) using an Olympus Bx51 microscope.

### Molecular detection of pathogens

#### DNA extraction

Before DNA extraction, each ectoparasite was cut in half with a sterile blade. They were then incubated with buffer and Proteinase K at 56°C for 24 hours. Total DNA was extracted from blood (*n* = 48) and ectoparasites (*n* = 80) using the QIAamp DNA mini kit (Qiagen, Hilden, Germany) according to the manufacturer’s instructions. All samples were stored at −20°C until further analysis.

#### DNA preamplification for microfluidic PCR

To improve the detection of pathogens, a pre-amplification of total DNA was performed. For amplification, an equal volume of primers (except those targeting tick DNA and controls) was pooled at a final concentration of 200 µM. The total volume of the mixture was 5 µL, containing 1 µL of Perfect PreAmp Master Mix (Standard Biotools, San Francisco, CA), 1.25 µL of pooled primer mixture, 1.5 µL of distilled water, and 1.25 µL of DNA. The following program was used for DNA amplification: one cycle at 95°C for 2 minutes, 14 cycles at 95°C for 15 seconds and 4 minutes at 60°C. The samples were then diluted to 1/10 (addition of 45 µL of distilled water). The pre-amplified samples were stored at −20°C until further use.

#### Microfluidic PCR

High-throughput microfluidic real-time PCR was used to detect bacterial and parasitic pathogens. The total number of pathogens targeted was 27 bacterial species (*Borrelia burgdorferi* s.s., *B. garinii*, *B. afzelii*, *B. valaisiana*, *B. lusitaniae*, *B. spielmanii*, *B. bissettii*, *B. miyamotoi*, *Anaplasma marginale*, *A. platys*, *A. phagocytophilum*, *A. ovis*, *A. centrale*, *A. bovis*, *Ehrlichia canis*, *N. mikurensis*, *Rickettsia conorii*, *R. slovaca*, *R. massiliae*, *R. helvetica*, *R. aeschlimannii*, *R. felis*, *Bartonella henselae*, *Francisella tularensis*, *Francisella*-like endosymbionts, *Coxiella*-like endosymbionts, and *Coxiella burnetii*), five bacterial genera (*Borrelia*, *Anaplasma*, *Ehrlichia*, *Rickettsia*, and *Mycoplasma*), seven piroplasm species (*Babesia microti*, *B. canis*, *B. ovis*, *B. bovis*, *B. caballi*, *B. venatorum*, and *B. divergens*), and three higher piroplasm taxa (Apicomplexa, *Theileria*, *Hepatozoon*) (82, 132). Amplification was performed using 48.48 Dynamic Array IFC chips (Standard Biotools) in the BioMark real-time PCR system (Standard Biotools). In each chip, 48 samples were analyzed by real-time PCR against 48 targets in individual wells, resulting in 2,304 individual reactions. All amplifications were performed using 6-carboxyfluorescein (FAM)- and Black Hole Quencher (BHQ1)-labeled TaqMan probes with TaqMan Gene Expression Master Mix according to the manufacturer’s instructions (Applied Biosystems, Courtaboeuf, France). RT-PCR cycling program was as follows: 2 minutes at 50°C, 10 minutes at 95°C, followed by 40 cycles of two-step amplification of 15 seconds at 95°C, and 1 minute at 60°C. A negative water control was used for each chip. In addition, the DNA of *Escherichia coli* strain EDL933 was used for each sample as an internal inhibition control, together with primers and probes specific for the *E. coli eae* gene (82, 132).

#### Validation of microfluidic PCR results and DNA sequencing

Positive samples for Apicomplexa, *Bartonell*a spp., *N. mikurensis*, and *Mycoplasma* spp. were selected for additional conventional and nested PCR assays targeting different genes or using different primers of the BioMark real-time PCR system. We paired the positive blood samples with those that were positive for at least one ectoparasite (tick or bat fly). We used primers for the 18S rRNA gene of Apicomplexa (12) and the 16S rRNA gene of *Anaplasma* spp. (123), *Bartonella* spp. (133), and *Mycoplasma* spp. (134) (Table 3). Positive samples were purified and sent to Eurofins MWG Operon (Ebersberg, Germany) and Macrogen (Macrogen Europe, Amsterdam, The Netherlands) for sequencing. The sequences were further analyzed and assembled using BioEdit software (Ibis Biosciences, Carlsbad, Germany). All sequences obtained from bat samples were submitted to the GenBank database under the following accession numbers: OQ054993–OQ055014; OQ255847; OP999371–OP999375; OQ274904–OQ274910 (Table 1).

**TABLE 3:** Set of primers used to validate microfluidic real-time PCR results

Pathogen/pathogens targeted	Target gene	Name and primer sequence (5′–3′)	Amplicon size	Reference
*Anaplasma* spp./*Ehrlichia* spp.	16S rRNA	EHR1 (GAACGAACGCTGGCGGCAAGC) EHR2 (AGTAYCGRACCAGATAGCCGC)	-	123
		EHR3 (TGCATAGGAATCTACCTAGTAG) EHR4 (AGTAYCGRACCAGATAGCCGC)	629 bp	
Apicomplexa	18S rRNA	BTH-1F (CCTGAGAAACGGCTACCACATCT) BTH-1R (TTGCGACCATACTCCCCCCA)	-	135
		GF2 (GTCTTGTAATTGGAATGATGG) GR2 (CCAAAGACTTTGATTTCTCTC)	561 bp	
*Bartonella* spp.	*glt*A	bart781 (GGGGACCAGCTCATGGTGG) bart1137 (AATGCAAAAAGAACAGTAAACA)	380–400 bp	136
*Mycoplasma* spp.	16S rRNA	Myco184-F1 (ACCAAGSCRATGATRGRTAGCTGG) Myco1310-R1 (ACRGGATTACTAGTG ATTCCAACT TCAA)	-	137
		Myco322-F2 (GCCCATATTCCTACGGGAAGCAGCAGT) Myco938-R2 (CTCCACCACTTGTTCAGGTCCCCGTC)	500 bp	138

#### Phylogenetic and genetic distance analysis

To determine the identity and genetic diversity of each pathogen examined in this study (targeting 16S rRNA for *Mycoplasma*, *Bartonella*, Anaplasmataceae and 18S rRNA for Apicomplexa), the sequences obtained were analyzed using the Basic Local Alignment Search Tool (BLAST; https://blast.ncbi.nlm.nih.gov/Blast.cgi, accessed on 10 March 2023). After identifying each sequence at the species level, a search was performed analyzing sequences of the same bacterial/piroplasm species with different hosts which were previously identified in different regions of the world. Sequences within the same bacterial/piroplasm species with different hosts were grouped for phylogenetic analysis. A second search was conducted comparing our sequences with others that were closely related or not at the species level and grouped for a second phylogenetic analysis. Each grouped sequence was then aligned with MUSCLE algorithm in MEGA 11 (139). To construct initial phylograms, the maximum parsimony, neighbor joining, and maximum likelihood (ML) methods were used. Due to their similar topology, ML was used in the final analysis. Based on the lowest Bayesian Information Criterion and corrected Akaike Information Criterion, Kimura 2-parameter model was used to build the trees of Anaplasmataceae and *Bartonella* (comparing sequences at species level), while Jukes-Cantor model was applied to construct the host-species tree. Tamura 3-parameter model (T92) was used to build trees of Apicomplexa. Reliability of internal branches was assessed using the bootstrapping method with 500 replicates.

The sequences shown in the trees were grouped into haplotypes (genotypes) using the DnaSP software (Universitat de Barcelona, Spain, http://www.ub.edu/dnasp). To show the genetic diversity of the bacteria/piroplasms depending on the reservoir hosts, the Median Joining Network method available in POPArt software (University of Otago Popart, https://popart.maths.otago.ac.nz) was applied.

## Data Availability

All the data sets shown in the present study can be offered on request.

## SUPPLEMENTAL MATERIAL

Fig. S1 spectrum.01531-23-s0001.tif Phylogeny of Anaplasmataceae inferred from 16S rRNA.

Fig. S2 spectrum.01531-23-s0002.tif Phylogeny of Apicomplexa inferred from 18S rRNA.

Fig. S3 spectrum.01531-23-s0003.tif Phylogeny of *Mycoplasma* sp. inferred from 16S rRNA.

Fig. S4 spectrum.01531-23-s0004.tif Phylogeny of *Bartonella* sp. inferred from 16S rRNA.

Fig. S5 spectrum.01531-23-s0005.tif Phylogeny and genotype analysis of Anaplasmataceae inferred from 16S rRNA depending on host reservoir species.

Fig. S6 spectrum.01531-23-s0006.tif Phylogeny and genotype analysis of Apicomplexa inferred from 18S rRNA depending on host reservoir species.

Supplemental Table S1 spectrum.01531-23-s0007.xlsx Summary of PCR and sequencing results.

ASM does not own the copyrights to Supplemental Material that may be linked to, or accessed through, an article. The authors have granted ASM a non-exclusive, world-wide license to publish the Supplemental Material files. Please contact the corresponding author directly for reuse.
