# Supplementary figures and images for: Detection of bacterial and protozoan pathogens in individual bats and their ectoparasites using high-throughput microfluidic real-time PCR
Source: Microbiol Spectr. 2023 Aug 22;11(5):e01531-23. doi: 10.1128/spectrum.01531-23 (PMC10581248; doi:10.1128/spectrum.01531-23)

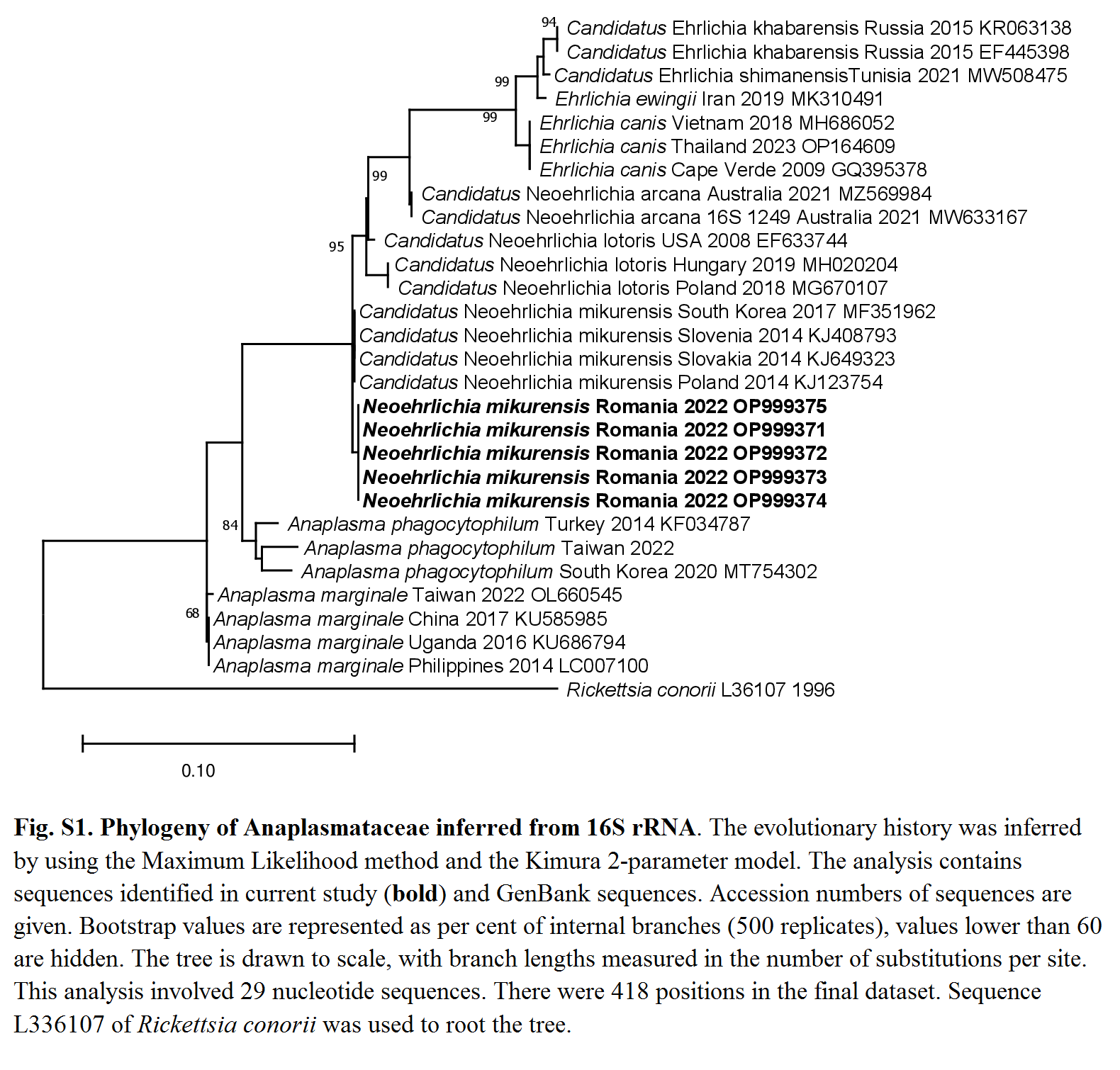

Supplement: Fig. S1 — Phylogeny of Anaplasmataceae inferred from 16S rRNA. [file spectrum.01531-23-s0001.tif]

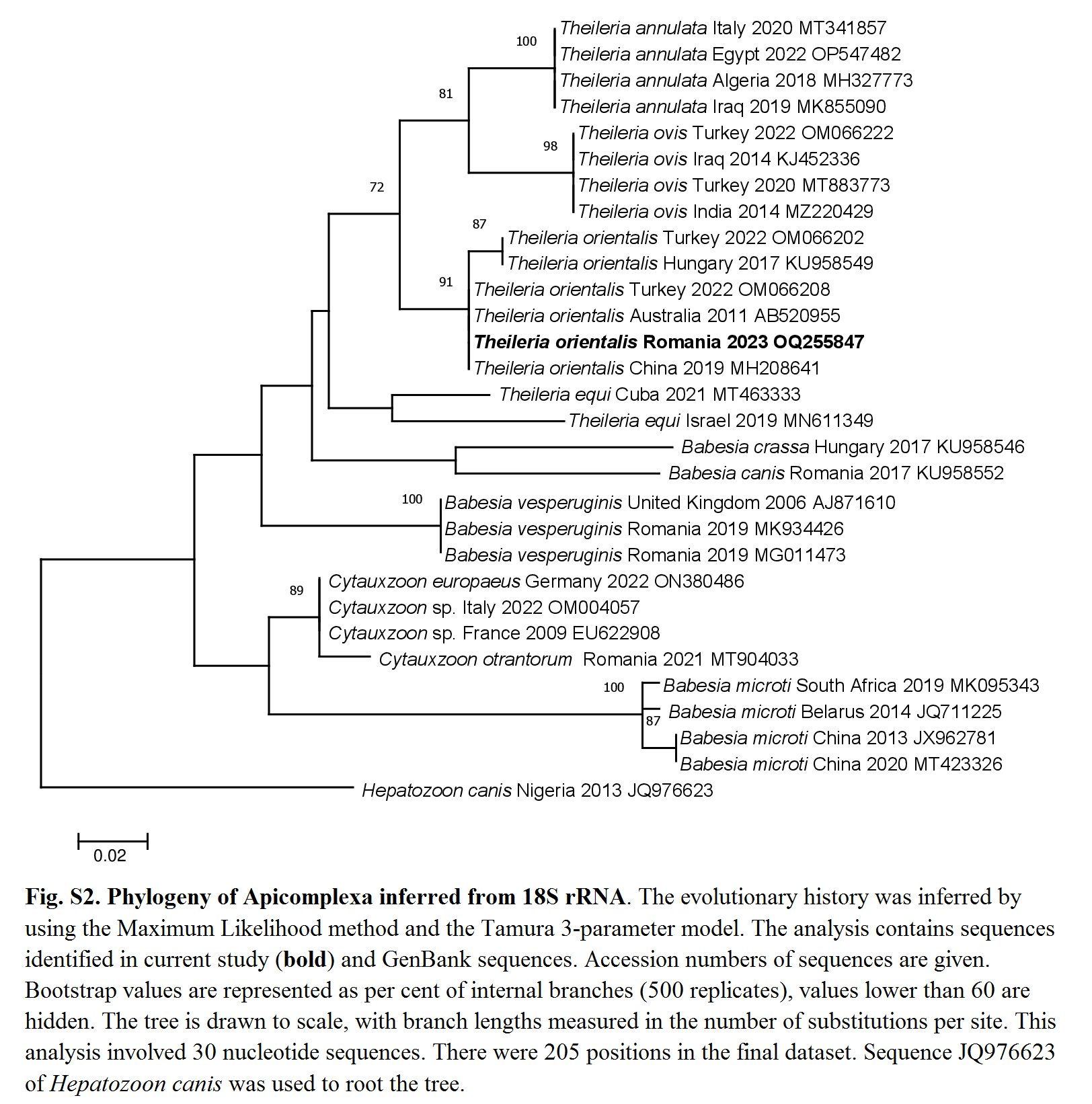

Supplement: Fig. S2 — Phylogeny of Apicomplexa inferred from 18S rRNA. [file spectrum.01531-23-s0002.tif]

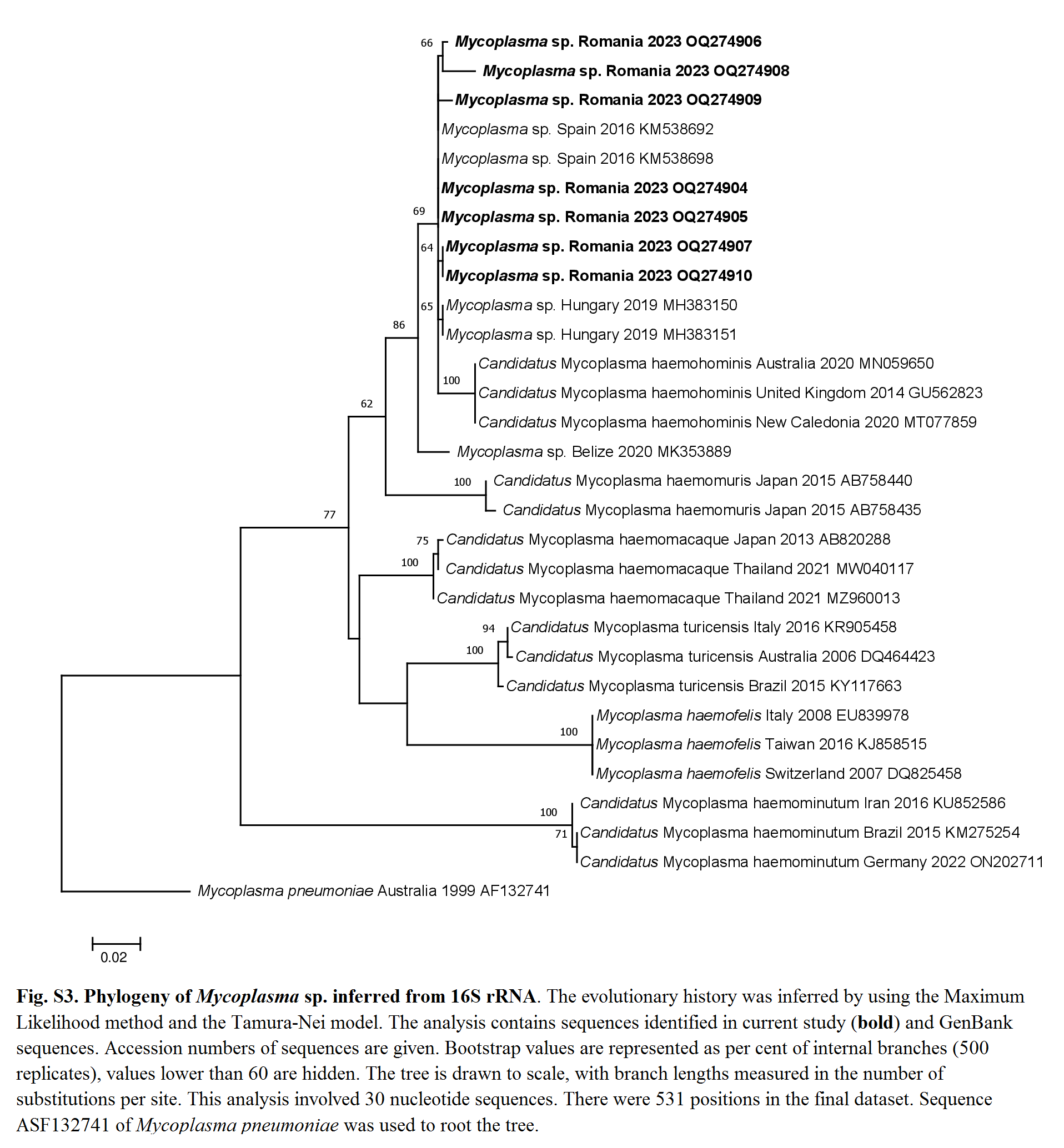

Supplement: Fig. S3 — Phylogeny of Mycoplasma sp. inferred from 16S rRNA. [file spectrum.01531-23-s0003.tif]

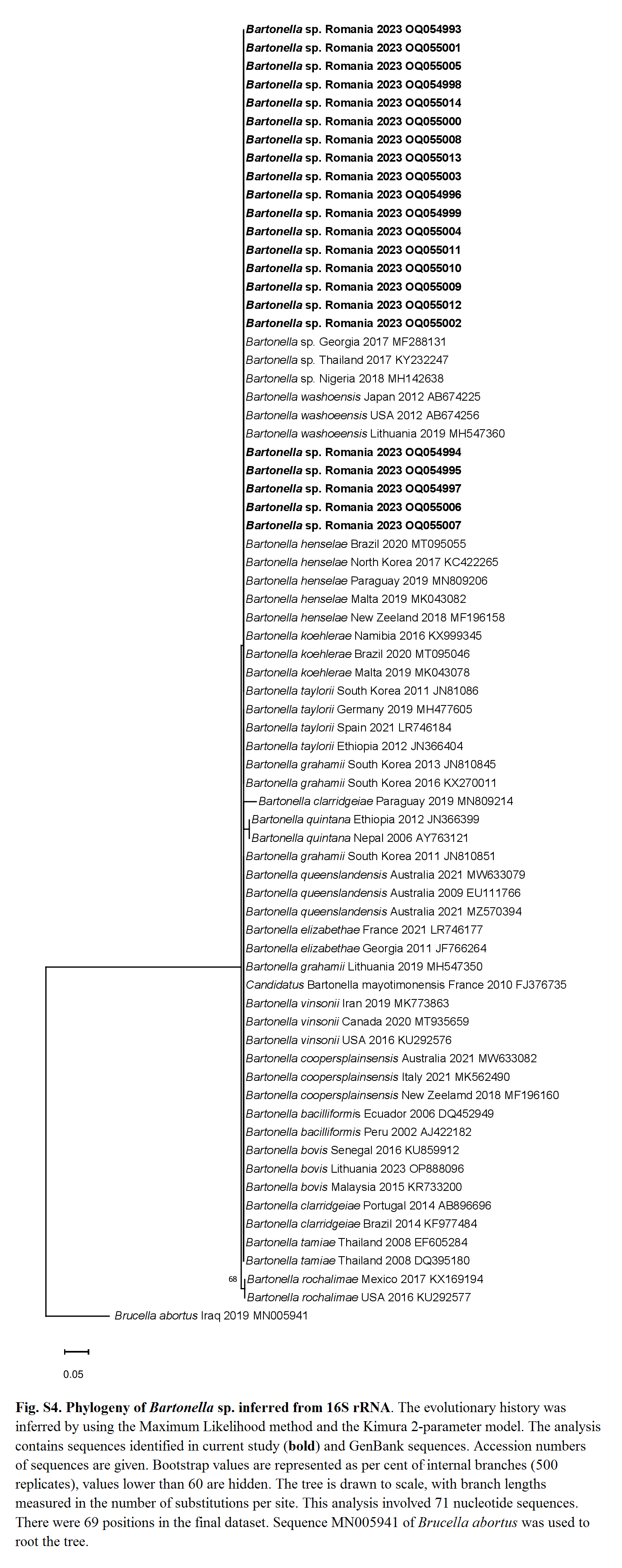

Supplement: Fig. S4 — Phylogeny of Bartonella sp. inferred from 16S rRNA. [file spectrum.01531-23-s0004.tif]

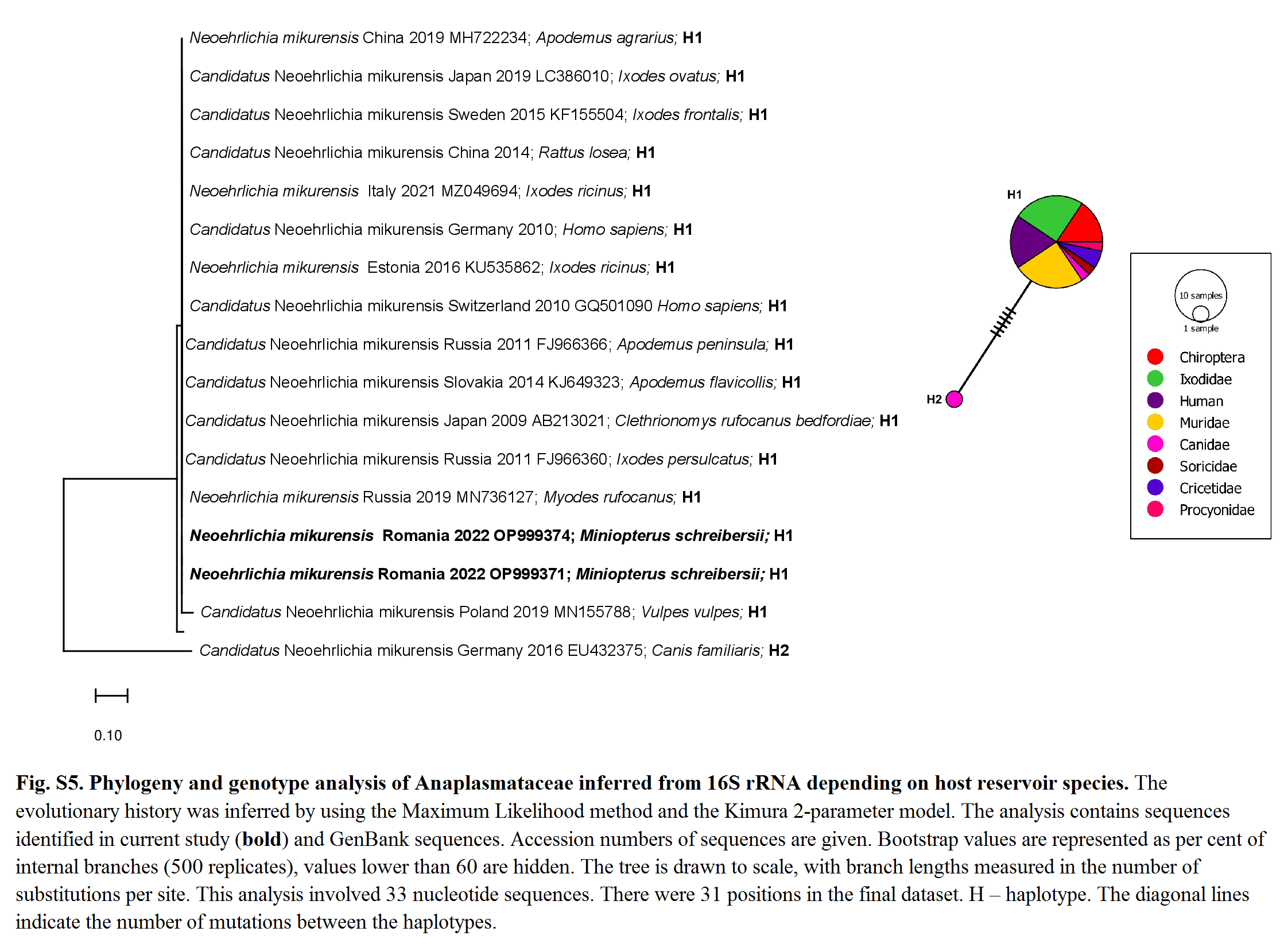

Supplement: Fig. S5 — Phylogeny and genotype analysis of Anaplasmataceae inferred from 16S rRNA depending on host reservoir species. [file spectrum.01531-23-s0005.tif]

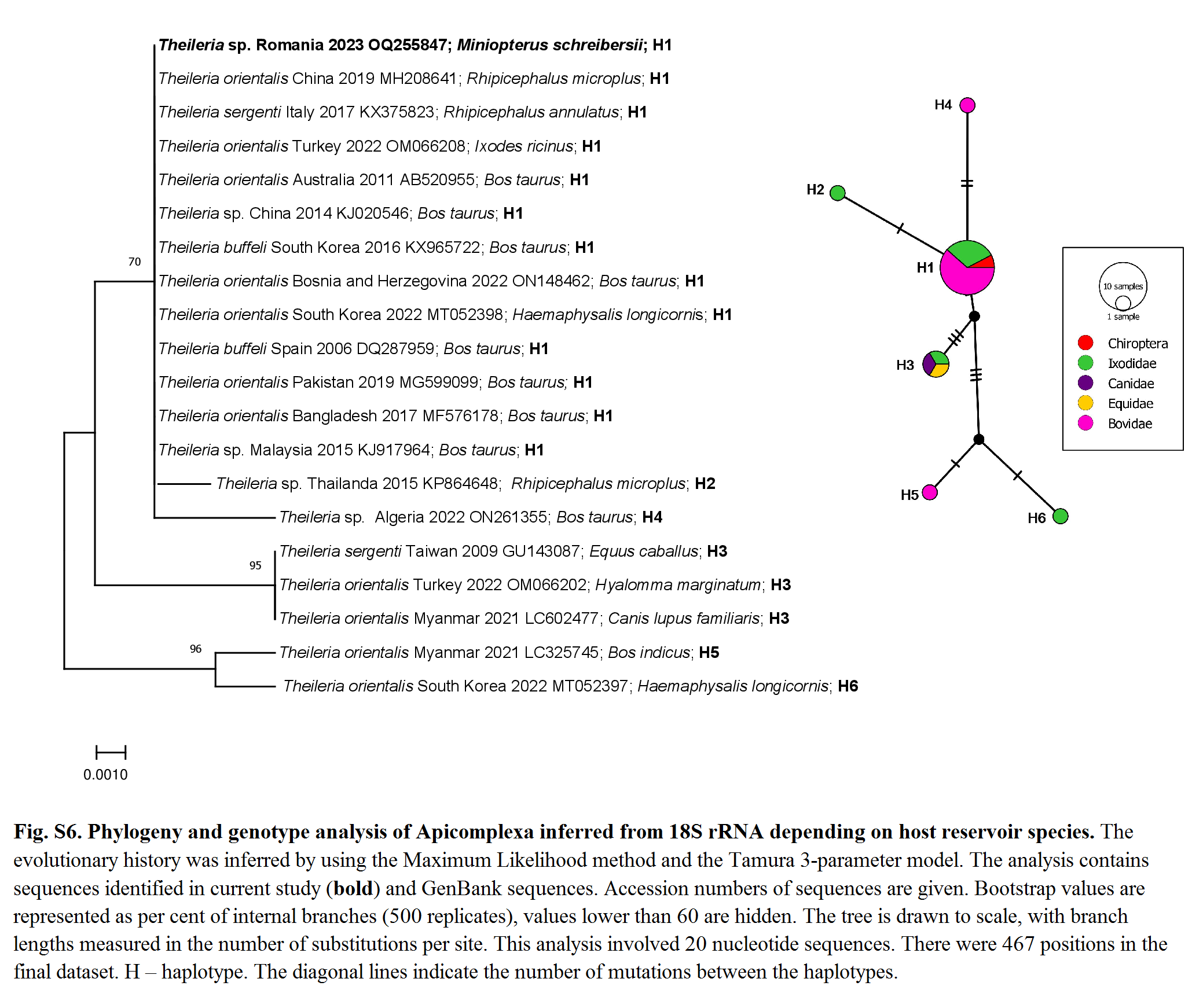

Supplement: Fig. S6 — Phylogeny and genotype analysis of Apicomplexa inferred from 18S rRNA depending on host reservoir species. [file spectrum.01531-23-s0006.tif]
